# Development and evaluation of 16 new microsatellite loci for the rock ptarmigan (*Lagopus muta*) and cross-species amplification for the willow grouse (*L. lagopus*)

**DOI:** 10.1186/s13104-018-3249-1

**Published:** 2018-02-20

**Authors:** Jean-Marc Costanzi, Frode Bergan, Mona Sæbø, Andrew Jenkins, Øyvind Steifetten

**Affiliations:** Department of Natural Sciences and Environmental Health, University College of Southeast Norway, Gullbringvegen 36, 3800 Bø i Telemark, Norway

**Keywords:** Birds, *Lagopus muta*, *Lagopus lagopus*, Microsatellites, Population genetics

## Abstract

**Abstract:**

The genetic markers designed for this study can facilitate future genetic studies on the rock ptarmigan (*Lagopus muta*). To our knowledge no microsatellite markers have ever been developed specifically for this species before. These new microsatellite markers will be useful for population genetics studies and for future conservation projects.

**Results:**

Using Next Generation Sequencing 6252 potential microsatellite sequences were found. Sixteen nonpalindromic tetranucleotide microsatellites and their respective primers were selected. The markers were tested on both the rock ptarmigan and the willow grouse (*L. lagopus*). The number of alleles varied between 2 and 18 for the rock ptarmigan, and between 3 and 13 for the willow grouse. Expected heterozygosity was in the range 0.1244–0.8692 and 0.1358–0.8722 for the rock ptarmigan and the willow grouse, respectively.

## Introduction

The rock ptarmigan (*Lagopus muta*) and the willow grouse (*L. lagopus*) are two closely related bird species that inhabit the alpine zone throughout the northern hemisphere. In Scandinavia they are important game species. Both have experienced a drastic decline in recent years [1] and there is an urgent need for tools to investigate the underlying causes of the decline, including markers for population genetic studies. To our knowledge only two studies on population genetics have been carried out on the rock ptarmigan, but none of the markers used were species-specific [2, 3].

Microsatellite markers have many applications [4], and with only a limited number of markers needed, they are among the most popular type of genetic markers used in ecological studies [5]. Although single-nucleotide polymorphisms (SNPs) are becoming increasingly popular for population genetic studies, due to the ease with which they can be read, microsatellites still have some advantages [5]. Microsatellite markers are more polymorphic than SNPs [6], so a smaller number of loci is needed for the same level of precision [7], decreasing the cost of the analysis. Moreover some studies suggest that microsatellite markers are better than SNPs to detect recent population structure events [8, 9]. However, in order to prevent high scoring error rates, only long motifs (tetranucleotides), which are less subject to slippage, should be selected [10]. This also aids inter-laboratory comparison.

To avoid the problem of null alleles, polymerase chain reaction (PCR) based markers require primer sequences that bind to highly conserved regions with few or no inter-individual differences. To minimize errors due to primer site mutations it is best to use species-specific markers [11].

The number of markers required is inversely correlated with the degree of genetic differentiation across populations. Thus, for large populations or species with a high migration rate (as is the case for the rock ptarmigan), a larger number of microsatellites is required [4]. In this study, we have designed microsatellite markers specifically for the rock ptarmigan and also tested their potential with the closely-related willow grouse.

## Main text

### Materials and methods

For the library construction approximatively 1 mg of ethanol-preserved liver sample from a rock ptarmigan originating from the western part of the Hardangervidda plateau, Norway, was used. This was sent to Cornell University (Ithaca, New York, USA), where the following analyses were conducted as a commercial service. At the Evolutionary Genetics Core Facility a library was prepared; this was then sent to the Sequencing and Genotyping Facility (Cornell Life Sciences Core Laboratory Center) for Titanium 454 sequencing. The sequences obtained were assembled under stringent condition with a match size of 120 base pair (bp), a match percentage of 94% and a minimum sequence length of 150 bp. From this dataset all di-, tri-, tetra-, penta- and hexanucleotides repeats were screened with the software *msatcommander* [12, 13] and sequences less than 100 bp and/or with a small number of repeats were excluded. Primers were designed with Primerselect software (part of the Lasergene package [14]). Every microsatellite sequence chosen was run through the online version of the Basic Local Alignment Search Tool for nucleotide (BLASTn) [15] in order to check for similarity with already published sequences.

To examine whether the selected microsatellites exhibited enough polymorphism, additional rock ptarmigan samples were collected from two distinct areas 324 km apart (Table 1). DNA from tested populations came from feathers (from hunted birds), and from fecal pellets found on the ground [16].

**Table 1:** Summary of the genetic samples collected in southern Norway and in Sweden during 2015/16

Site	Species	No. individuals	Fecal pellets	Feathers	Latitude	Longitude
Bykle (Norway)	Rock ptarmigan	32	6	26	59° 25′ 23.95″	7° 3′ 50.49″
Lesja (Norway)	Rock ptarmigan	31	NA	31	62° 10′ 22.55″	9° 0′ 9.55″
Ringebu (Norway)	Willow grouse	21	21	NA	61° 34′ 21.27″	10° 20′ 9.74″
Fulufjället (Sweden)	Willow grouse	22	22	NA	61° 29′ 15.86″	12° 40′ 31.51″

DNA extraction, PCR and genotyping of the tested populations was done at the genetic laboratory of the University College of Southeast Norway. Two different DNA extraction kits were used depending on the type of sample; the “DNeasy Blood & Tissue kit” (Qiagen, Cat. No. 69506) for feathers, and the “QIAamp DNA Stool Mini Kit” (Qiagen, Cat. No. 51504) for fecal pellets. In order to increase the quantity of DNA extracted during the “DNeasy Blood & Tissue” procedure, 5 µL of DTT 1 M was added to the ATL buffer during the first step of the protocol. In the “QIAamp DNA Stool Mini Kit” protocol, the quantity of buffer ASL was increased from 1.4 to 1.6 mL. In order to assess the efficiency of DNA extraction, the concentration of DNA was measured with a Qubit™ dsDNA HS Assay kit (Thermo Scientific™ Cat. No. Q32854) on a Qubit 3.0 Fluorometer (Thermo Scientific™) for 30 samples. Each sample was amplified with species-specific mitochondrial DNA primers Lagsp3F, Lag3R and Mut3R [see 17] in order to distinguish *L. muta* and *L. lagopus*, whose fecal pellets are not distinguishable morphologically, and whose habitats overlap. PCR conditions follow Nyström et al. 2006 [17], and the results were read via gel electrophoresis [16]. Rock ptarmigans were identified by an amplicon size of 212 bp, and willow grouse by an amplicon size of 154 bp.

For microsatellite analysis, PCR amplification was performed with forward primers labeled with FAM NED, PET or VIC fluorescent dyes. The reaction was made with 6µL of “Qiagen Taq PCR mastermix” (Qiagen, Cat. No. 201445) with a final concentration of 1×, 0.25 µL of a 20 µM stock of each primers, 2 µL of DNA template and ultrapure water to a total volume of 12 µL. PCR was done in an Eppendorf Mastercycler^®^ gradient thermal cycler: 15 min at 95 °C, 40 cycles consisting of 30 s at 94 °C, 90 s at 60 °C, 60 s at 72 °C, and finally 10 min at 72 °C. Microsatellite markers were multiplexed in five different PCR reactions. For every reaction a negative control was included and analysed in parallel with the samples. If any positive signal was found in the negative control the results were rejected and the entire run was repeated. In order to check for consistency of the data, 28 rock ptarmigan samples were tested twice.

For the genotyping 1.5 µL of PCR product was added in a mix containing 9.7 µL of formamide (Thermo Scientific™, Cat. No. 17899) and 0.3 µL of GeneScan 500LIZ dye Size Standard (Applied Biosystems™, Cat. No. 4322682). Genotyping was realized on a 3130xl Genetic analyzer (Applied Biosystems). Allele scoring was done using GeneMapper software V5.0 and visually controlled (Applied Biosystems). To avoid analyzing the same individual twice, an individual identity analysis was carried out with the software Cervus V3.0.7 [18]. The minimum number of matching loci was set to 7, and 1 fuzzy matching was allowed. Microsatellite markers were then tested for the presence of null alleles with the software *microchecker* [19]. Linkage disequilibrium and Hardy–Weinberg equilibrium were tested with the software GENEPOP [20]. In order to control for multiple testing, the *p* value threshold was adjusted with Bonferroni correction. Number of alleles *N*a, observed heterozygosity *H*_o_ and expected heterozygosity *H*_e_ were calculated with the software Genetix V4.05 [21, see Table 2]. The same procedures were also performed on samples from the willow grouse, which were collected from two different areas 124 km apart in Norway and in Sweden (Table 1).

### Results

After the Titanium 454 sequencing 31037 sequences were obtained, which resulted in 6252 potential microsatellite sequences after screening with *msatcommander*. Sixteen microsatellite nonpalindromic tetranucleotide repeats with lengths between 85 and 226 bp, and with number of repeats between 5 and 15 were selected. BLASTn analysis of the sequences gave two positive matches with red jungelfowl (*Gallus gallus)* sequences. The first sequence named CH261-100D18 (Accession Number AC147597) had an 87% match with Mut18, but the repeat motif was not present on the *Gallus galllus* sequence. The second sequence named 8F6 (Accession Number X78623.1 [22]), had a 90% match with Mut01 and the repeat motif was identical. Because we couldn’t find any publications using this microsatellite on grouse, we kept it for analysis.

In total 63 rock ptarmigan individuals were tested (Table 1). Measured DNA quantity for the 30 samples tested ranged from 0.06 to 44 ng/µL (average: 7 ng/µL). Identity analysis with Cervus detected three redundant individuals that were removed prior to analysis. Replicate data was available for 28 rock ptarmigan samples, representing a total of 371 pairs of allele identification (failed reaction were not counted). There was in total nine mismatches between replicates, one mismatch for Mut18, Mut4 and Mut22, two mismatches for Mut12 and three mismatches for Mut20. The software *microchecker* detected one microsatellite marker (Mut12) that presented a high frequency of null alleles. No significant linkage disequilibrium was found after Bonferroni-correction. A significant deviation (p < 0.0031) from Hardy–Weinberg (heterozygosity deficit) was found after Bonferroni correction on markers Mut01 and Mut12. The total number of alleles per microsatellite ranged from 2 (Mut24) to 18 (Mut17). Expected heterozygosity was between 0.1244 (Mut24) and 0.8332 (Mut08), and observed heterozygosity ranged between 0.1333 (Mut24) and 0.8571 (Mut04) (Table 2).

**Table 2:** Characterization of 16 polymorphic microsatellites for the rock ptarmigan (regular font) and the willow grouse (italics)

Locus name	Repeat motif of cloned allele	GenBank accession number	Primer sequence (5′–3′)	Primer dye	Allele size range (bp)	*N*a	*H*_E_	*H*_O_
Mut01	(ATCC)8	MF425753	F: GGTCACTTGTGGCTATTAGAACC	PET	85–104	6	0.6545	0.6129 [0.0016]**
			R: CTGAGAAAGACAATGGTGGATGG		*85*–*115*	*8*	*0.7361*	*0.6667* [*0.0606*]
Mut02	(ATCC)9	MF425754	F: GATGGATGAACAGACAGTGGATG	FAM	99–127	8	0.6454	0.6984 [0.7326]
			R: CACTCACTTGCTTATATGATTCCC		*103*–*127*	*6*	*0.7243*	*0.5366* [*0.0001*]***
Mut03	(AAAC)7	MF425755	F: TCACTAAATCATGGAAAGCAATGG	NED	100–116	5	0.6925	0.7069 [0.8376]
			R: GGTGAGGTGGATTATCATATGCAG		*NA*	*NA*	*NA*	*NA*
Mut04	(ATCC)11	MF425756	F: AACATGGTCCTACACTTCAGAGG	VIC	107–131	6	0.7602	0.8571 [0.9064]
			R: ATGTACACAAGGAAGCATTCAGG		*115*–*151*	*8*	*0.8324*	*0.8421* [*0.2652*]
Mut06	(AAAC)7	MF425757	F: TTGAACACTAATCTCGCCATTGC	PET	110–130	6	0.6983	0.6984 [0.5816]
			R: GTGGGATATGAGGAAAGAGTTGC		*114*–*126*	*4*	*0.5541*	*0.2093* [*0.0001*]***
Mut08	(AAGC)6	MF425758	F: TACTACTTTCTGAACTCTGCTGC	VIC	122–176	9	0.8332	0.7705 [0.0032]*
			R: GAGAGAAGAAGGAACAAACAAGC		*122*–*201*	*11*	*0.8722*	*0.7105* [*0.0037*]*
Mut09	(AGAT)10	MF425759	F: CACTCTAGTTCAACCTGTTCAGC	VIC	125–161	8	0.8083	0.7931 [0.3421]
			R: CTCTTAGAGAATTTGCTGCTGTG		*125*–*153*	*8*	*0.8072*	*0.4250* [*0.0000*]***
Mut12	(AAAC)6	MF425760	F: GGAAGGAGCTTACACATAGGAAC	NED	123–150	7	0.7448	0.5932 [0.0000]**
			R: GGAGGATCTTGTACTTGCAGTTG		*146*–*154*	*6*	*0.5971*	*0.5814* [*0.4043*]
Mut14	(AAAC)5	MF425761	F: TCTGCCAACTTCTTTATGCTGTC	FAM	154–174	6	0.6596	0.6825 [0.4565]
			R: TTCTTTCAATTCATTAGCCCAGTG		*148*–*160*	*4*	*0.1358*	*0.0952* [*0.0478*]*
Mut16	(AGAT)12	MF425762	F: TGCTGTAATTGGCTAGTGGTTTC	NED	155–171	5	0.6658	0.6452 [0.4086]
			R: CGGTCAGGTGTTTCTCAGTAAAG		*151*–*183*	*9*	*0.8164*	*0.7209* [*0.0275*]*
Mut17	(AAAG)15	MF425763	F: CCAAGAACACAGAAATCCCAGAG	PET	134–226	18	0.7586	0.6207 [0.0105]*
			R: TGTTAATTGACTGCCACAAACTAC		*126*–*174*	*5*	*0.4207*	*0.3514* [*0.1756*]
Mut18	(AAAC)6	MF425764	F: TTCACTCATAACTAAGCAAAGCAG	FAM	171–183	4	0.6122	0.5690 [0.0747]
			R: TGCATTGGAATTACTGTTGATGC		*178*–*183*	*3*	*0.4436*	*0.4048* [*0.3302*]
Mut20	(ATCC)13	MF425765	F: CTTTCCACCTTTCTTACTGCCTG	PET	173–213	11	0.8692	0.7500 [0.0068]*
			R: CTCATCCTGTATTTCTGAGCTGC		*157*–*217*	*13*	*0.8430*	*0.7143* [*0.0344*]*
Mut22	(AAAC)6	MF425766	F: GTGGTATCTCTGTAACTTGGGAG	FAM	179–195	3	0.4259	0.4444 [0.6885]
			R: AAGGCAAAGCAAGAATGTCTGTG		*179*–*191*	*4*	*0.6684*	*0.6098* [*0.2344*]
Mut23	(AGAT)9	MF425767	F: CTGGAGTCTAGAATTGCCACAAC	NED	173–209	10	0.8206	0.7833 [0.1645]
			R: AGGGTCAAGAACTTACAATGGAC		*173*–*197*	*7*	*0.7064*	*0.7027* [*0.3735*]
Mut24	(AAAG)5	MF425768	F: AACTGACAATCAAGCATCTCATG	PET	198–202	2	0.1244	0.1333 [1.0000]
			R: CTGCTATGTACTACTCAAGGTGC		*NA*	*NA*	*NA*	*NA*

NA, no available data for the species; *N*a, number of observed alleles; *H*_O_, observed heterozygosity; *H*_E_, expected heterozygosity

Brackets: p values for the exact tests of departure from Hardy–Weinberg proportions (H1 heterozygosity deficit)

* p < 0.05, ** p < 0.0031 (Bonferroni correction for the rock ptarmigan), *** p < 0.0033 (Bonferroni correction for the willow grouse)

For the willow grouse the microsatellite markers Mut03 and Mut24 were hard to read and therefore removed from analysis, but the other microsatellites produced amplicon of size and pattern equivalent to the rock ptarmigan. Two microsatellite markers, Mut06 and Mut09, presented a high frequency of null alleles. No significant linkage disequilibrium was found after Bonferroni-correction. A significant deviation (p < 0.0033) from Hardy–Weinberg (heterozygosity deficit) was found after Bonferroni correction for markers Mut02, Mut06 and Mut09. The total number of alleles per microsatellite for the willow grouse ranged from 3 (Mut18) to 13 (Mut20), expected heterozygosity from 0.1358 (Mut14) to 0.8722 (Mut08), and observed heterozygosity from 0.0952 (Mut14) to 0.8421 (Mut04).

### Discussion

Most of the tested microsatellite markers produced good results, except for Mut01 and Mut12, which both presented a heterozygosity deficit. The heterozygosity deficit of Mut01 could be explained by the Wahlund-effect [23], as we pooled two different populations together for the Hardy–Weinberg equilibrium calculation. When calculating heterozygosity deficit separately for each of the two populations, only Mut12 presented a significant heterozygosity deficit after Bonferroni correction. The significant heterozygosity deficit for Mut12 is probably due to the presence of null alleles.

The need for high quality and species-specific microsatellite markers resulted in the development of a new library for the rock ptarmigan. The microsatellites have primers optimised for the target species and are designed to have a low amount of stutter, by selecting tetranucleotide repeat motifs, which are significantly less prone to slippage and a repeat number of five in order to achieve high degrees of polymorphism and heterozygosity, [24]. The high level of heterozygosity and the important number of alleles makes them suitable for studies on population structure and/or dispersal behaviour.

## Limitations

- The number of published microsatellite markers could be too low for population structure analysis at a local scale.
- While tetranucleotide motif are less prone to slippage, they are also less polymorphic than di- or trinucleotide motif.
- These microsatellite markers have only been tested on two species of grouse.

## Abbreviations

PCR: polymerase chain reaction
bp: base pair
BLASTn: Basic Local Alignment Search Tool for nucleotide
Na: number of alleles
H_o_: observed heterozygosity
H_e_: expected heterozygosity
